# Oligodendrocyte-specific overexpression of human alpha-synuclein results in elevated MBP levels and inflammatory responses in TgM83 mice, mimicking the pathological features of multiple system atrophy

**DOI:** 10.1186/s40478-025-02014-y

**Published:** 2025-05-07

**Authors:** Sam Chi-Hao Liu, Koping Chang, Meng-Ling Chen, Ming-Che Kuo, Teh-Cheng Wang, Ruey-Meei Wu

**Affiliations:** 1 Department of Neurology, National Taiwan University College of Medicine, No.1, Jen Ai Road section 1, Taipei City, Taiwan (R.O.C.); 2 Department of Neurology, National Taiwan University Hospital, No.7, Chung Shan South Road, Taipei City, Taiwan (R.O.C.); 3 Department of Pathology, National Taiwan University Hospital, No.7, Chung Shan South Road, Taipei City, Taiwan (R.O.C.); 4 Department of Medicine, National Taiwan University Cancer Center, No.57, Ln. 155, Keelung Road Section 3, Taipei City, Taiwan (R.O.C.)

**Keywords:** MBP, AAVOlig001, TgM83 mice, Alpha-synucleinopathy, Inflammation responses, MSA

## Abstract

Multiple system atrophy (MSA) is a neurodegenerative disorder characterized by parkinsonism, cerebellar dysfunction, and autonomic failure. Key pathological features of MSA include the formation of glial cytoplasmic inclusions (GCIs) in oligodendrocytes (OLs), myelin loss, and neuroinflammation. Although both inflammation and myelination are known to be critical in MSA, the roles of myelin proteins and their relationship with inflammation have often been overlooked. In this study, we injected AAV-Olig001 vectors carrying either human *SNCA* (AAV-hSNCA) or eGFP (AAV-eGFP) into the striatum of TgM83 transgenic mice, which express the A53T mutant form of human alpha-synuclein (αSyn), as well as into wild-type (WT) mice. We then assessed myelin protein expression and inflammatory responses. TgM83 mice injected with AAV-hSNCA exhibited demyelination, increased activation of microglia and astrocytes, and altered cytokine and chemokine profiles (including IL-1α, IL-10, IL-12(p40), CCL2, CCL3, CCL4, and CCL5), compared to both WT mice and TgM83 mice injected with AAV-eGFP. Interestingly, myelin basic protein (MBP) levels were significantly elevated around the injection site in TgM83 mice injected with AAV-hSNCA. Notably, we observed a positive correlation between MBP expression and inflammatory markers, as indicated by Iba1 and GFAP staining. These findings suggest that hSNCA overexpression is associated with increased MBP levels and enhanced inflammatory responses, implicating that MBP and myelination processes may play previously underappreciated roles in the pathogenesis of MSA.

**Supplementary Information:**

The online version contains supplementary material available at 10.1186/s40478-025-02014-y.

## Introduction

Multiple system atrophy (MSA) is a rare neurodegenerative disease that is characterized by neuronal loss and gliosis in multiple areas of the central nervous system including striatonigral, olivopontocerebellar and central autonomic structures. It is fatal, rapid progressive and currently has no treatment. Thus, it’s important to understand the pathogenesis of MSA and the mechanisms that are associated with it.

Recent studies using MSA patients’ serum [28], cerebrospinal fluid (CSF) [11, 50, 53], and post-mortem brain tissues [26, 46] suggested that excessive neuroinflammation is closely associated with MSA pathologies. For instance, Compta and colleagues reported that 12 of the 38 tested cytokines were significantly increased (FGF-2, CCL-11, fractalkine, INF-a2, IL-10, CCL-7, IL12-p40, CCL-22, IL-17, IL-6, MIP-1β, and TNFα) in CSF in MSA compared to non-MSA cases including patients with Parkinson’s disease (PD) and health controls [11]. Starhof and colleagues examined a panel of C-reactive protein (CRP) and cytokines among patients with PD, MSA, progressive supranuclear palsy (PSP) and health controls. They found that CSF levels of CRP, TNF-α, IL-1β, and IL-6 were significantly elevated in MSA and PSP patients compared to PD but not controls [50]. Furthermore, neuroinflammation in putaminal white matter regions of MSA patients coincided with elevated numbers of aSyn inclusions, while gray matter with less α-synucleinopathy remained unaffected [26]. Widespread microglial activation was also found in the early stage of MSA, particularly in those with parkinsonian type [30].

One of the histopathological hallmarks of MSA is glial cytoplasmic inclusions (GCI), mainly composed of alpha synuclein (αSyn) phosphorylated at serine-129 residue (pS129 αSyn) [20], in oligodendrocytes [42]. Cell loss and GCIs are initially restricted to stratum (putamen, caudate) and substantia nigra, then gradually involved subthalamic nucleus and dentate gyrus, followed by other regions in basal ganglia and midbrain, like globus pallidus and tectum. In the end, the pathology will reach lower brainstem, cerebellum, and spinal tract [13, 22]. The decreased levels of dihydroxyphenylacetic acid, the main neuronal metabolite of dopamine, had also been demonstrated in the CSF of MSA patients [48]. Accompanied by the accumulation of GCI in oligodendrocytes, demyelination has also been identified as one of the pathological features of MSA, as shown in post-mortem tissue [17] and animal models [12, 34, 52]. Counterintuitively, recent studies showed that the number of oligodendrocyte progenitor cells (OPC) or oligodendrocytes was increased in the white matter region of MSA patients [2] and animal models [17, 39]. One of the myelin proteins, myelin basic protein (MBP), was significantly elevated in the CSF of MSA patients compared to PD and controls [47]. These findings suggest that the myelination process may be impaired in MSA.

Despite inflammation and the myelination are two important processes in MSA, the changes of myelin proteins in the MSA pathology and the relationship between myelin proteins and inflammation is often overlooked. In this study, we tried to develop a MSA mouse model with particular interest on the changes of myelin proteins. We aimed (1) to develop a MSA mouse model with abundant abnormal αSyn aggregation in oligodendrocytes, (2) to study the inflammation responses that are associated with αSyn aggregation, and (3) to analyse the changes of myelin proteins and its relationship with inflammation responses. While previous studies only applied the viral vector on wide-type (WT) mice, we injected AAVOlig001 carrying hSNCA into transgenic mice expressing A53T human αSyn line (TgM83). This provides a genetic background for abnormal αSyn aggregation and synucleinopathy hence it is expected to enhance the potency of αSyn induced pathologies.

## Materials and methods

### Mice

To investigate whether the pre-existing abnormal αSyn could aggravate the αSyn-induced pathologies, both WT and TgM83 mouse were used in this study. Homozygous and hemizygous TgM83 mice (B6; C3-Tg(Prnp-SNCA*A53T)83Vle/J; 004719) were purchased from the Jackson Laboratory and bred on an B6; C3 background. C3H/HeNCrNarl (C3H, no. RMRC11004) and C57BL/6JNarl (B6, no. RMRC11005) were purchased from National Laboratory Animal Center in Taiwan. C57BL/6 x C3H mice were generated by crossing female B6 mice with male C3 mice. All mice were maintained under pathogen-free conditions with a 12/12 h light/dark cycle, 20–25 °C with 60% relative humidity and free access to food and water. All animal experimental procedures were approved by the Institutional Animal Care and Use Committee of National Taiwan University and carried out in accordance with the guidelines of the Committee, and the “Principles of laboratory animal care” (NIH publication No. 86 − 23, revised 1985) were followed.

### AAVOlig001 vector

Olig001 is a special viral serotype which was generated by using capsid shuffling and directed evolution and could exhibit a > 95% tropism for striatal oligodendrocytes after rat intracranial infusion [43]. In mixed glial cell cultures, Olig001 exhibited a 9-fold greater binding capacity than AAV8 [43]. The AAV vectors were produced by the University of North Carolina Vector Core facility by triple-transfecting production plasmids into HEK293 cell [52]. Two vector constructs were utilized: Olig001/PscAAV-CBh-FLAG-EGFP (AAVeGFP; qPCR titer vg/ml = 1 × 10^12) and Olig001/PscAAV-CBh-FLAG-hSNCA (AAVhSNCA; qPCR titer vg/ml = 7.6 × 10^11); both dialysed in 350mM NaCl + 5%D-Sorbitol in 1xPBS. 3x FLAG tag sequence was also attached to distinguish endogenous proteins from exogenous proteins.

### Stereotaxic injection surgery

Two-month-old male and female mice were used in this study to model the physiology of young adult humans, aligning with established practices in MSA and Parkinson’s disease mouse models [23, 52]. Under isoflurane anesthesia, mice received a unilateral injection of 2 µL into the dorsolateral striatum. Animals were randomly assigned to receive one of the following: saline (control group), AAVeGFP (sham control group), or AAVhSNCA (experimental group). Injections were administered at a rate of 0.5 µL/min using a 25 µL Hamilton Neuros syringe (Model 1702 RN, 33-gauge, Point Style 4). Upon completion of the injection, the needle remained in place for an additional 3 min to minimize backflow before being slowly withdrawn. Stereotaxic coordinates relative to bregma were: anterior-posterior (AP) + 0.5 mm, medial-lateral (ML) − 2.0 mm, and dorsal-ventral (DV) − 2.7 mm from the dura [52].

### Immunofluorescence (IF) of mouse samples

To examine molecular alterations between the injected and non-injected hemispheres, immunostaining was performed to assess changes in myelin-associated proteins, inflammatory markers, pS129 αSyn, and neuronal density.

At two months post-injection, mice were deeply anesthetized with isoflurane and transcardially perfused with PBS followed by 4% paraformaldehyde (PFA). Brains were extracted, post-fixed in 4% PFA at 4 °C for 72 h, and then cryoprotected in 30% sucrose in PBS until fully submerged. The brains were embedded in Tissue-Tek^®^ O.C.T. Compound and stored at − 80 °C. Coronal Sect. (14 μm thick) were prepared using a freezing microtome and processed for immunofluorescence analysis. Sections were washed and incubated in 5% donkey serum for 1 h at room temperature to block nonspecific binding. Primary antibodies were applied at a 1:200 dilution and incubated overnight at 4 °C.

The antibodies for myelin proteins: Anti-MBP clone SMI 99, purified, BioLegend; Anti-myelin oligodendrocyte glycoprotein (MOG) Antibody, MAB5680, Sigma-Aldrich (Merck); Anti-myelin proteolipid protein (PLP) antibody, ab28486, Abcam. For inflammatory responses: Recombinant Anti-Iba1 antibody [EPR16588] (ab178846), Abcam; GFAP Monoclonal Antibody (GA5), eBioscience™, 14-9892-82, Thermofisher (Invitrogen); VEGF Monoclonal Antibody (VG1), MA1-16629, Invitrogen. VEGF was used as an indirect biomarker for inflammation because of the strong connection between inflammation and angiogenesis [3, 4]. For αSyn aggregates: Recombinant Anti-Alpha-synuclein (phospho S129) antibody [EP1536Y] (ab51253), Abcam; Anti-TPPP antibody [EPR3316] (ab92305), Abcam. For neurons: Anti-NeuN antibody [EPR12763] - recombinant Alexa Fluor^®^ 647, Abcam.

After primary antibody incubation, sections were washed and incubated with the following secondary antibodies for 1 h at room temperature: Donkey anti-Rabbit IgG (H + L), Alexa Fluor™ 488, and Donkey anti-Mouse IgG (H + L), Alexa Fluor™ 546; both Highly Cross-Adsorbed, Thermo Fisher. Nuclei were counterstained with DAPI and mounted using Fluoromount-G^®^ (0100 − 20, Southern Biotech). Fluorescent images were acquired using a Zeiss Axio Observer 7 fluorescence microscope with 10× or 20× objectives and captured digitally for subsequent quantitative analysis.

### Luxol fast blue (LFB) staining for demyelination

LFB was used for identifying myelin and demyelination. Sections were immersed in 95% alcohol for 5 min, then immersed in 0.1% Luxol Fast Blue solution at 58–60 °C overnight, followed by 95% alcohol again. Sections were stained using 0.05% lithium carbonate solution and 70% alcohol. Sections were then counterstained with Hematoxylin for 1 min and Eosin for 30 s, followed by 95% alcohol. Sections were then dried and cover slipped.

### LFB, neuron, Myelin proteins, microglia and astrocyte imaging quantification (mice)

LFB fold change in the striatal injection side was measured relative to the non-injection side. Images were contrast-enhanced using CorelDRAW 2021 and then exported for analysis in ImageJ (version 1.54f). All images were converted to 16-bit grayscale, and mean gray values were measured on both sides. Since ImageJ assigns a pixel value of 255 to white and 0 to black, images were inverted to accurately capture staining intensity. Fixed-size ROIs were applied consistently across all sections. Relative intensity was calculated by dividing the mean gray value of the injection side by that of the non-injection side. A total of 3–5 animals per group were quantified.

Neuron numbers were determined using NeuN staining and quantified with StrataQuest software. Since NeuN is localized to the nucleus, only nuclear signals were detected. The threshold for identifying NeuN-positive cells was automatically set by StrataQuest. Regions of interest (ROIs) were defined based on pS129 α-synuclein staining, and only areas showing pS129 α-synuclein expression were selected for analysis. The density of NeuN-positive stained cells (neurons per mm²) on the injection side was used to assess the differences, *n* = 3–6 animals were quantified per group.

To assess changes in myelin-associated proteins, immunofluorescence images of MBP, MOG, and PLP were analyzed using ImageJ software (v1.54f). Only sections with detectable pS129 αSyn signal were selected. Identically sized ROIs were placed over the affected regions in both hemispheres. The mean fluorescence intensity (mean gray value) was measured using ImageJ, and relative expression levels were calculated as the ratio of the injected side to the non-injected side. A total of *n* = 4–8 animals per group were used for quantification.

Microglial and astrocyte activation was assessed via Iba1 and GFAP staining, respectively. Since the contralateral side has scant Iba1 and GFAP signals, only signals from the ipsilateral (injection) side were used for analysis. Given that activation is accompanied by cellular morphological changes, the percent area occupied by stained cells within defined ROIs was used as a surrogate measure of activation. ROIs were positioned in regions showing the highest signal intensity (see Fig. 3), and only areas with confirmed pS129 α-synuclein positivity were analyzed. All ROIs were of uniform size. Images were converted to 8-bit, and thresholding was manually adjusted. The built-in “Analyze Particles” function in ImageJ (v1.54f) was used to quantify signal area. A total of *n* = 3–5 animals per group were quantified.

**Fig. 1:**
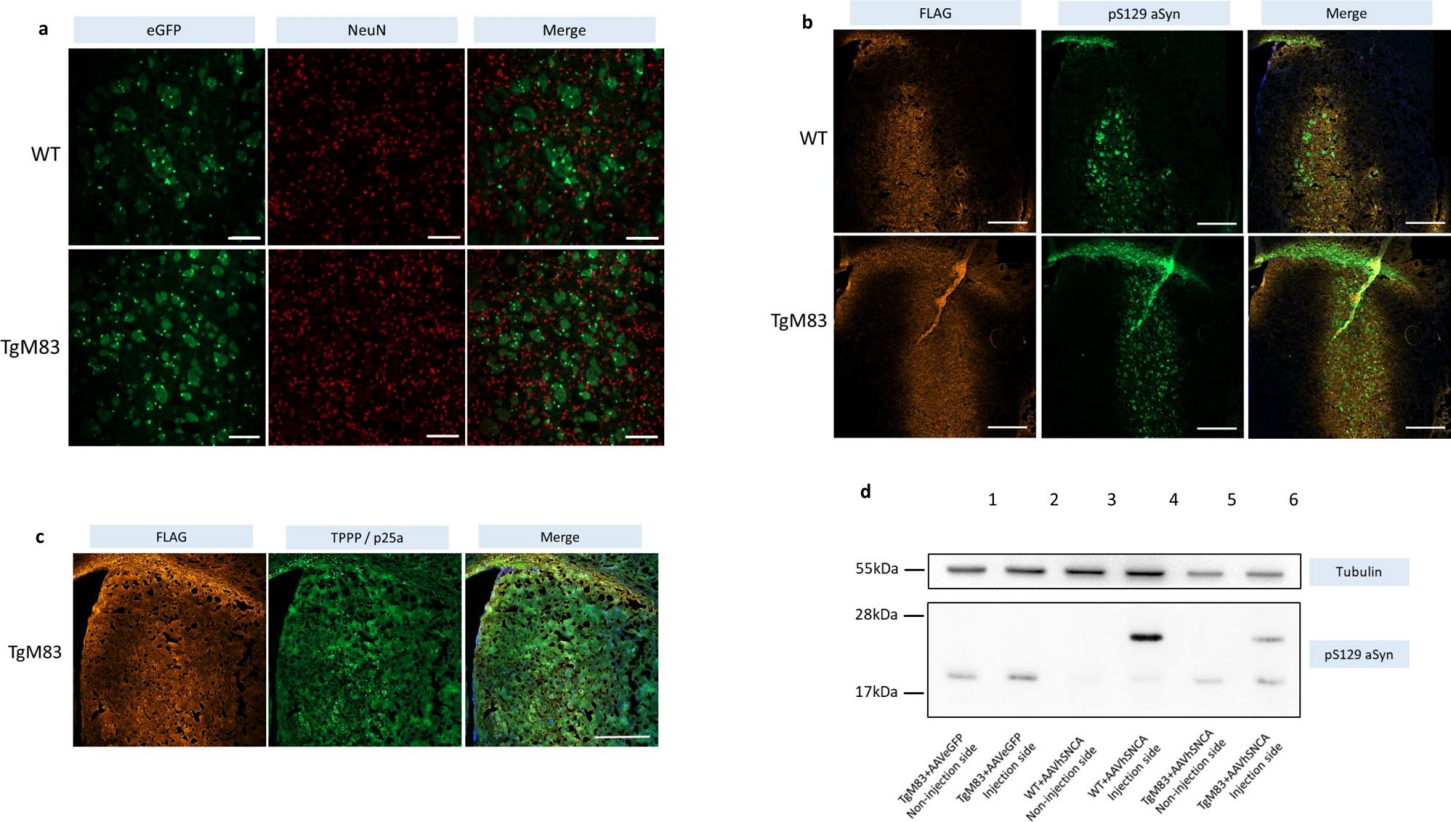
AAVOlig001 can induce exogenous pS129 αSyn overexpression in non-neuronal cells in the injected region. **a** AAVeGFP signals do not co-localize with neuron signals in both WT and TgM83. The majority of eGFP signals (green) do not co-localize with neuron signals (NeuN, red), suggesting preferential oligodendrocyte tropism. Scale bar = 100 μm. **b** AAVhSNCA was injected into WT and TgM83. pS129 αSyn overexpression (green) was confined within the FLAG expressing regions (orange). Scale bar = 500 μm. **c** TPPP/p25a signals also overlap the FLAG-stained region. Scale bar = 500 μm. **d** Exogenous and endogenous pS129 αSyn were shown in WT and TgM83 by WB. The endogenous pS129 αSyn are generated by TgM83 mice as shown in line 1, 2, 5, 6). Exogenous pS129 αSyn can be seen in the injection side of WT and TgM83 mice (line 4, 6) (The uncropped gels and blots are available in Supplementary Fig. 1)

**Fig. 2:**
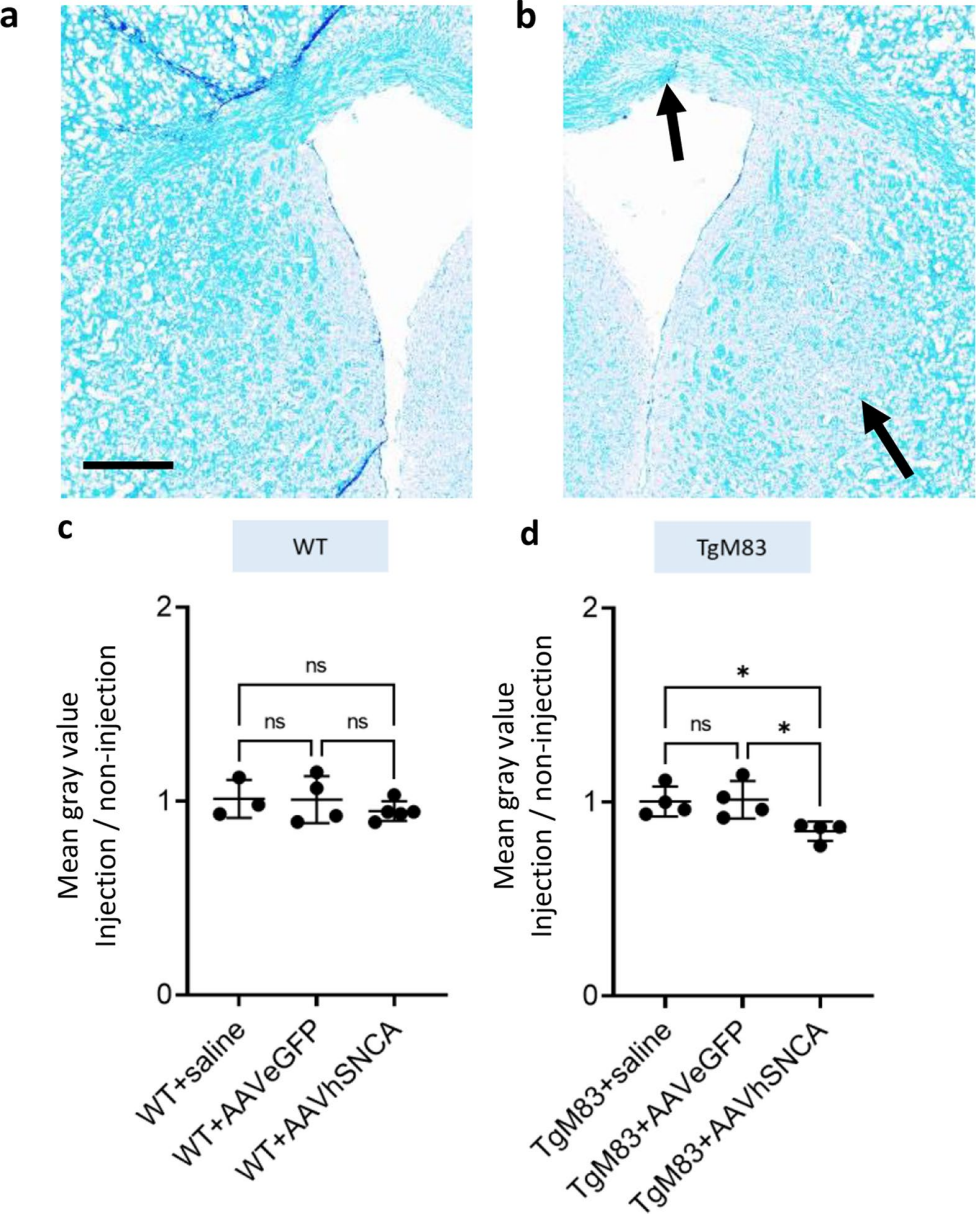
Regional demyelination was observed in the injection side in TgM83 + AAVhSNCA group. **a**,** b** LFB shows areas with demyelination in the injection side of TgM83 + AAVhSNCA (arrow). Decreased LFB staining was shown on the injection side in TgM83 + AAVhSNCA, but not on the non-injection side, Scale bar = 0.5 mm. **c**,** d** LFB staining revealed demyelination in the injection side of TgM83 + AAVhSNCA, but not in WT + AAVhSNCA. Myelin is quantified as fold change relative to the contralateral (non-injection) side in WT and TgM83

### Immunohistochemistry (IHC) of mouse samples

To quantify CD4⁺ T cells in the mouse striatum, IHC was performed using the CONFIRM anti-CD4 (SP35) rabbit monoclonal antibody (catalog no. 05552737001, Roche). CD4⁺ cells modulate both innate and adaptive immune responses through distinct phenotypes and cytokine profiles [33]. Automated staining was conducted using the BenchMark ULTRA IHC/ISH System (Roche Diagnostics, USA). Tissue sections were imaged with a TissueFAXS system (TissueGnostics) using a 20× objective. Only nucleated cells approximately 10 μm in diameter were counted as CD4-positive. A total of *n* = 3 animals were included for quantification.

### Transmission electron microscopy

TEM was used to look at the myelin structure in the striatum of a TgM83 mice injected with AAVhSNCA. Mouse sections were fixed using 2.5% glutaraldehyde for 2 h in 4 °C. The tissue was then dehydrated with graded series of ethanol solutions (50%, 75%, 85% and 95%) for 10 min each; then in 100% ethanol 2 times for 20 min each. The sections were photographed and examined under a transmission electron microscope (JEM-1400; JEOL USA Inc., Peabody, MA, USA).

### Western blot (WB) and quantification

WB was used to quantify the expression level of pS129 αSyn and MBP. The mouse brains were first cut into 2–3 mm thick coronal sections using a rodent brain matrix. A small piece of striatum was then punched from the injection side and the non-injection side using a 2 mm Miltex biopsy punch.

Brain tissues were lysed in RIPA lysis buffer containing 25 mM Tris-HCl pH 7.6, 150 mM NaCl, 1% NP-40, 1% sodium deoxycholate, and 0.1% sodium dodecyl sulfate (SDS), supplemented with a protease inhibitor cocktail (Roche 4693116001) on ice for 30 min. Protein concentrations were then determined using the Bradford Assay (biorad 5000006). Thirty micrograms of protein per sample were mixed with 5X sample buffer and boiled for 10 min. The samples were then separated using 10% SDS polyacrylamide gels and transferred to PVDF membranes (Millipore, Billerica, MA, USA). The blotted membranes were blocked with 5% non-fat milk in Tris-buffered saline/0.2% Tween 20 (TBST) for 1 h at room temperature and incubated with primary antibodies [(Anti MBP, clone SMI 99, purified, BioLegend (1:5000) or recombinant anti-Alpha-synuclein (phospho S129) antibody [EP1536Y], Abcam ab51253 (1:5000)] overnight at 4 °C. Anti-HSP60, abcam 45134(1:10000) or tubulin, Genetex GTX101279 (1:10000) were used as internal controls. The membranes were then washed with TBST and incubated with horseradish peroxidase-conjugated secondary antibodies for 1 h at room temperature. Immunoreactive signals were detected using the chemiluminescent reagent ECL (wbkls0500, Millipore).

Quantification of the blots was analyzed using ImageJ 1.54f, where the intensity of each protein band was measured. WB bands on each lane were selected and plotted using image J, including MBP and HSP60. The values of each target protein / lane was normalized based on the values of the internal controls. The relative expression level was then calculated by dividing the value of the injection side by the non-injection side, 3 WT + AAVhSNCA and 5 TgM83 + AAVhSNCA animals were quantified.

### Bio-Plex multiplex immunoassays

Three mouse brains were first cut into 2–3 mm thick coronal sections using a rodent brain matrix. A small piece of striatum was then punched from the injection side and the non-injection side using a 2 mm Miltex biopsy punch. Cytokine and chemokine profiles induced by αSyn overexpression in striatum were analyzed by Bio-Plex multiplex immunoassays (Bio-Plex Pro Mouse Cytokine 23-plex Assay #M60009RDPD) according to the manufacturer’s protocol [35].

### MSA patient profile

Two MSA patients’ and controls’ brain tissues were obtained from the Department of Pathology, NTUH, and Taiwan Brain Bank Association. Case 1 is a 67-year-old male with MSA-P, disease duration of 3 years, with orthostatic hypotension and urinary retention. Case 2 is a 53-year-old male with MSA-C, disease duration of 4 years, with orthostatic hypotension and urinary retention, poor levodopa responsive Parkinsonism, and stridor. Control 1 is a 75-year-old male with aortic regurgitation, Mitral Regurgitation, Tricuspid Regurgitation, Status Post Operation and Pneumonia (AR, MR, TR s/p op; pneumonia) respectively. Control 2 is a 55-year-old female diagnosed of WHO grade IV gliosarcoma in the right frontal region at age of 54. She received right fronto-emporal craniotomy and radiotherapy. She was expired due to radionecrosis in the right front-temporal regions with cerebral edema one year later. Pathological change was not observed in bilateral basal ganglia.

### IHC of human samples

IHC is a common method for formalin-fixed paraffin-embedded (FFPE) staining to detect specific proteins. Same primary antibodies against phosphorylated αSyn at S129, Iba1 and MBP were used to look at the αSyn aggregation, inflammation, and changes in myelin proteins. Tissues are fixed in 10% buffered formalin and embedded in paraffin. Next, serial sections were cut (5 μm) using Leica RM2155. The slides were generated under routine sample preparation procedures using BenchMark ULTRA IHC/ISH System - Roche Diagnostics USA auto-staining machine. Stained sections were imaged with TissueGnostics TissueFAXS with 20x objective lens.

Image J was used to measure the intensity of MBP and Iba1 in the putamen of MSA patient and control. 27 sub-regions were selected from the putamen of each brain sample. Only the regions around the striatopallidal fibers (pencil fibers of Wilson) were selected. Images were first converted to 8-bit format, and a manually adjusted threshold was applied consistently across all images prior to analysis. The intensity of MBP expression was measured using the “Mean Gray Value” function in ImageJ, while microglial cell size was quantified using ImageJ’s “Analyze Particles” function.

### Statistical analyses

All the statistical analyses and graph designs were performed using GraphPad Prism v10.2. The results in column graphs represent the mean ± SD.

One-tailed, paired t-test was used to compare the concentrations of cytokines and chemokines between injection and non-injection sides. One-way ANOVA followed by multiple comparisons was employed for comparisons of the 3 groups. For all tests, a *p* value of 0.05 was considered to indicate significant difference between or among groups.

## Results

### AAVOlig001 vector tropism and pS129 ΑSyn overexpression in WT and TgM83 mice

We firstly want to demonstrate the characteristic of AAVOlig001 by using qualitative analyses. Figure 1a showed that the majority of eGFP signals did not co-localize with neurons (NeuN signals) in both WT and TgM83 mice. This suggested that the intracranial injection of AAVhSNCA or AAVeGFP could successfully avoid neurons in mice brains. This was in alliance with previous findings [43, 52]. The IF images (Fig. 1b) showed that pS129 αSyn could be successfully induced in the striatum of WT and TgM83 in the injection side.

Since the plasmid in AAVhSNCA vectors also carried 3x FLAG tag, we could use anti-FLAG antibody to mark the region infected by AAV, as shown in Fig. 1b. When mouse brain slices were stained with both FLAG and pS129 αSyn, the pS129 αSyn (green) signals overlapped with the FLAG marked region (orange) and did not exceed its boundaries, suggesting that the exogenous pS129 αSyn did not trigger sequential spreading of abnormal pS129 αSyn beyond the infected regions at the time of sacrifice (Fig. 1b). The sagittal sections also did not show any anterior or posterior spreading of pS129 αSyn to other brain regions (data not shown). Figure 1c also showed that TPPP/p25a signals overlap the FLAG stained region, suggesting that TPPP/p25a aggregates could also be induced by AAVhSNCA.

In Fig. 1d, the upper bands indicated the exogenous pS129 αSyn, which carried 3x FLAG sequence and were thus heavier. The lower bands were pS129 αSyn that were generated endogenous in TgM83 mice (line 1, 2, 5 and 6). The molecular weight of the endogenous pS129 αSyn is around ~ 19kDa [15], and the exogeneous pS129 αSyn is ~ 21.8 kDa (the 3x FLAG tag is ~ 2.8 kDa). Line 1 and 2 showed WB results of TgM83 mice injected with AAVeGFP. Only endogenous pS129 αSyn (the lower band) were seen in the injection and non-injection sides. In line 3 and 4, only exogenous pS129 αSyn (the upper band) was seen in WT + AAVhSNCA. Endogenous and exogenous pS129 αSyn were present in TgM83 + AAVhSNCA mice as shown in line 5 and 6 (Fig. 1d) (The corresponding uncropped gels and blots are provided in Supplementary Fig. 1). The data showed that the exogenous pS129 αSyn could successfully be expressed in WT and TgM83 mice when AAVhSNCA were injected (Fig. 1d), but pS129 αSyn did not transduce to other brain regions at the time of sacrifice (Fig. 1b).

### AAVhSNCA induce regional demyelination in TgM83 mice

Since demyelination is one of the pathological hallmarks of MSA, Luxol Fast Blue (LFB) staining was used to evaluate the myelin integrity in WT and TgM83 mice injected with saline, AAV-eGFP and AAV-hSNCA. The results displayed regional demyelination as shown by decreased LFB staining in striatum of TgM83 mice when AAVhSNCA was injected (Fig. 2a, b), but not when AAV-eGFP or saline was injected (Fig. 2d; WT + saline: 1.003 ± 0.07766; WT + eGFP: 1.012 ± 0.09628; WT + hSNCA: 0.8495 ± 0.04972; one-way ANOVA, *p* = 0.0259). The relative mean gray value represents the ratio of myelin intensity (as stained by LFB) on the injection side compared to the non-injection side. Demyelination could also be seen in the corpus callosum of TgM83 + AAVhSNCA (Fig. 2b, arrow). On the other hand, none of the WT groups displayed significant difference (Fig. 2c; WT + saline: 1.012 ± 0.098; WT + eGFP: 1.008 ± 0.121; WT + hSNCA: 0.9495 ± 0.051; one-way ANOVA, *p* = 0.5437). It suggested that although AAVhSNCA can effectively induce the overexpression of pS129 αSyn in both WT and TgM83, demyelination was only observed in TgM83 mice.

NeuN staining was used to evaluate the effects of αSyn accumulation on neuronal loss by measuring the number of neurons per mm^2^. However, one-way ANOVA revealed no significant differences among the groups in either WT (WT + saline, 667 ± 211; WT + AAVeGFP, 571.8 ± 86.2; WT + AAVhSNCA, 464.9 ± 249.3 no. /mm^2^; *p* = 0.3848) or TgM83 mice (TgM83 + saline, 647.1 ± 15.7; TgM83 + AAVeGFP, 611.9 ± 81.9; TgM83 + AAVhSNCA, 530.9 ± 117.9 no. /mm^2^; *p* = 0.2329) despite a decreasing trend is shown in TgM83 mice (Supplementary Fig. 2).

Since demyelination was found in TgM83 + AAVhSNCA, we further tested if the motor function was altered. Rotarod, pole-test and open field test were used to evaluate the mice’s motor functions. However, none of the behaviour tests showed significant difference (Supplementary Fig. 3).

### AAVhSNCA induced enhanced inflammation in TgM83

**Fig. 3:**
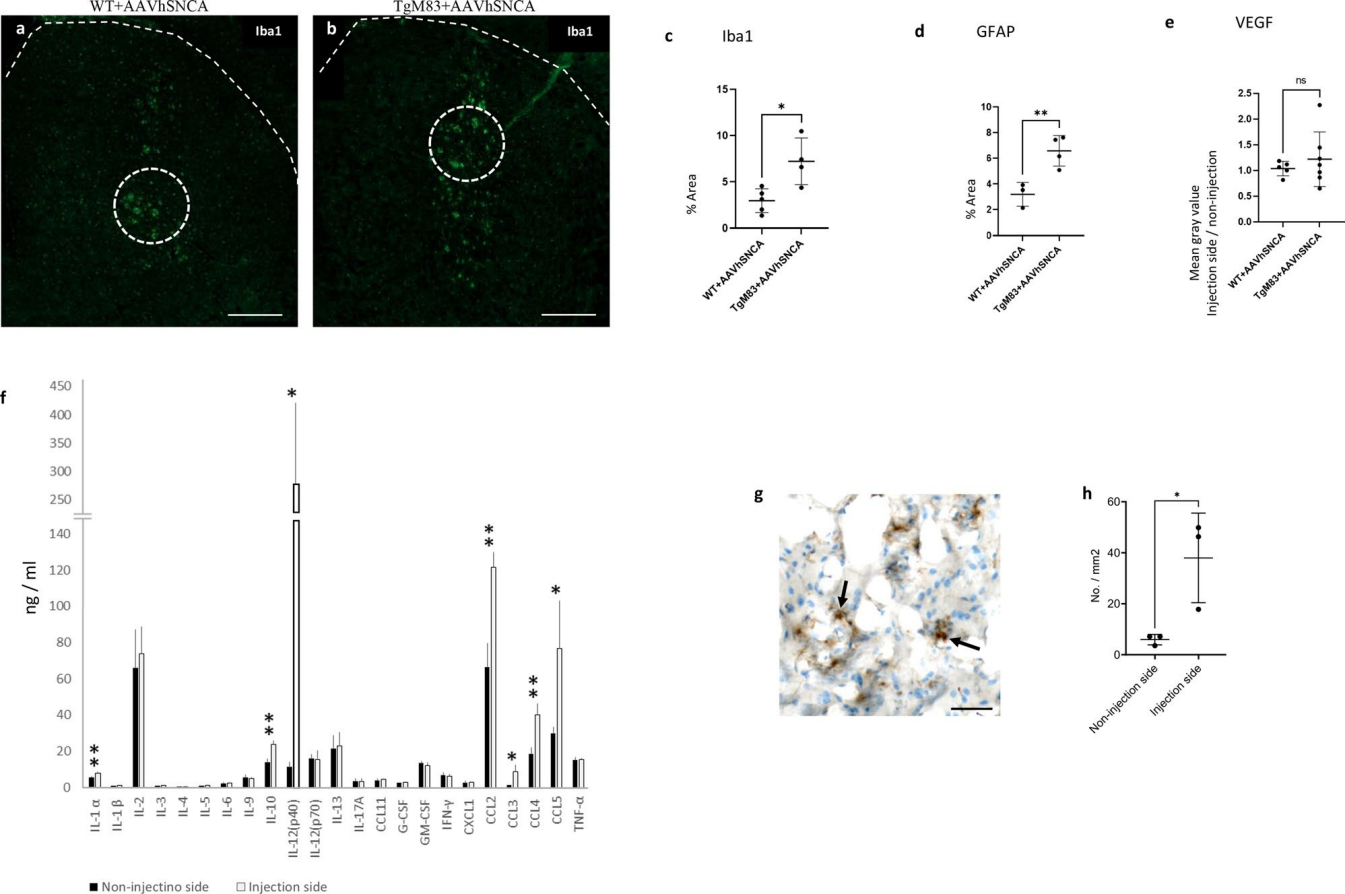
Inflammation responses were observed around the injection site in the striatum 2 months after AAVhSNCA injections. **a**,** b** IF images showed higher Iba1 activation (green) levels in the striatum of TgM83. Dotted lines mark the edge of striatum and ROI. Scale bar = 500 μm. **c**,** d** Iba1 and GFAP occupied higher area percentage in the selected region in TgM83, compared to WT. **e** VEGF staining released no difference between WT and TgM83. **f** Bio-Plex multiplex immunoassays showed that IL-1a, IL-10, IL-12(p40), CCL2, CCL3, CCL4, and CCL5 were increased in the injection side in TgM83 + AAVhSNCA, compared to the non-injection side. **g** CD4^+^ cells (arrow). Scale bar = 50 μm. **h** The increased number of CD4^+^ cells was found on the injection side following the injection of AAVhSNCA. * < 0.05; ** < 0.01

Since excessive inflammation is closely associated with MSA pathologies, we wanted to study the inflammation responses that are associated with αSyn aggregation in WT and TgM83 mice. TgM83 + AAVhSNCA showed higher Iba1% area, compared to WT + AAVSNCA (Fig. 3a-c, WT: 2.965 ± 1.286 vs. TgM83: 7.226 ± 2.522, unpaired t-test, *p* = 0.0128), suggesting a higher level of microglia activation or inflammation in TgM83 + AAVhSCA. GFAP stains also showed higher percent area in TgM83 + AAVhSNCA, compared to WT + AAVhSNCA (Fig. 3d, WT: 3.197 ± 0.935 vs. TgM83: 6.580 ± 1.187; unpaired t-test, *p* = 0.0098), suggesting a higher level of astrocyte activation or injury in TgM83 + AAVhSNCA. On the other hand, VEGF stains showed no difference between TgM83 + AAVhSNCA and WT + AAVhSNCA (Fig. 3e, WT: 1.037 ± 0.141 vs. TgM83: 1.219 ± 0.531; unpaired t-test, *p* = 0.4764), indicating that angiogenesis was not detected. These findings indicated a higher level of inflammatory responses in TgM83 + AAVhSNCA relative to WT + AAVhSNCA (Fig. 3).

In order to study the composition of cytokines involved in the inflammatory responses, Bio-Plex mouse cytokine 23-plex immunoassay was used to analyse small pieces of striatum that were dissected from both the non-injection and the injection side of the TgM83 + AAVhSNCA brains (*n* = 3). One-tailed, paired, t-test revealed elevated levels of IL-1a (*p* = 0.006), IL-10 (*p* = 0.005), IL-12(p40) (*p* = 0.029), CCL2 (*p* = 0.004), CCL3 (*p* = 0.027), CCL4 (*p* = 0.006) and CCL5 (*p* = 0.035) in the injection side (Fig. 3f) (detailed results are provided in Supplementary Table 1).

Since the elevated chemokines CCL2, CCL4, and CCL5 suggested the recruitment of immune cells, CD4^+^ cells were quantified using IHC (Fig. 3g, h). Two-tailed, unpaired, t-test revealed the increased number of CD4^+^ cells on the injection side, compared to the non-injection side (*p* = 0.03).

### AAVhSNCA induced MBP elevation in TgM83 mice, compared to WT animal

**Fig. 4:**
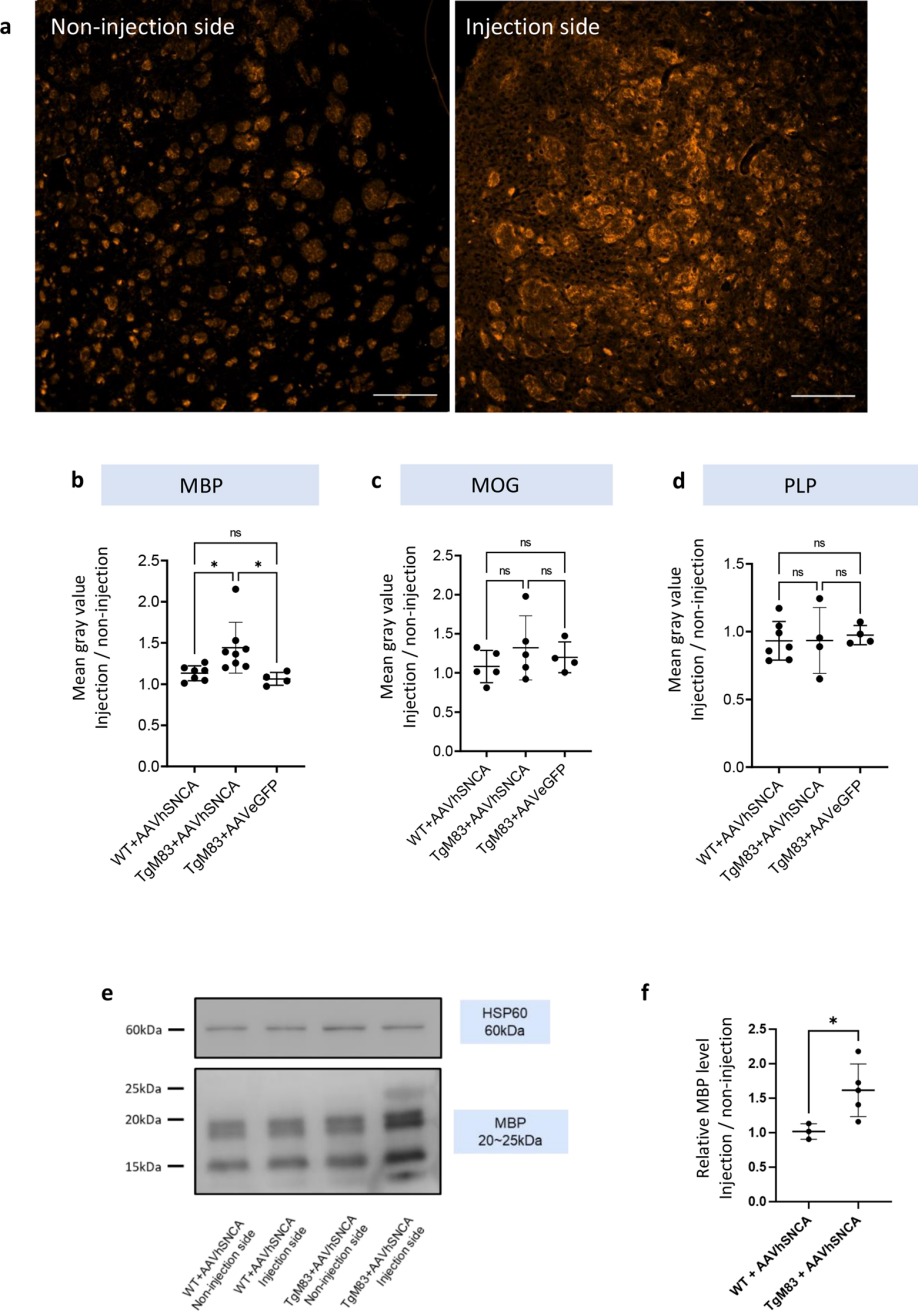
AVOlig001-hSNCA can successfully induce MBP overexpression in TgM83, but not WT. **a** IF images of MBP signals (orange) in TgM83 + AAVhSNCA mice. The elevated MBP levels was observed on the injection side. Scale bar = 200 μm. **b** Bar graph showed that MBP was greatly increased in TgM83 + AAVhSNCA, but not in WT + AAVhSNCA mice or when TgM83 + AAVeGFP was injected. **c**,** d** Other myelin proteins (MOG and PLP) did not reveal significant difference among all groups. **e** WB results showed that MBP was increased in the injection side of the TgM83 mice (line 4) (The uncropped gels and blots are available in Supplementary Fig. 1). **f** The fold change of MBP level between injection and non-injection side in WT + AAVhNSNCA and TgM83 + AAVhSNCA. The relative MBP level increased 1.62 times in TgM83 + AAVhNCA, compared to WT + AAVhSNCA

To investigate how αSyn overexpression could affect myelination process, the levels of myelin proteins (MBP, MOG and PLP) were analysed. The MBP signals was firstly measured using IF (Fig. 4a, b) and WB (Fig. 4e, f). The mean gray values of the MBP signal on both the injection and non-injection sides were measured, and the signal ratio was calculated for each mouse. Significant difference was found among groups (TgM83 + AAVeGFP: 1.064 ± 0.076; WT + AAVhSNCA: 1.131 ± 0.089; TgM83 + AAVhSNCA: 1.440 ± 0.309; *p* = 0.0131; one-way ANOVA) (Fig. 4b). IF data showed that MBP levels were 1.273 times higher in TgM83 + AAVhSNCA group compared to WT + AAVhSNCA group (*p* = 0.0331), and 1.353 times higher compared to TgM83 + AAVeGFP group (*p* = 0.0282). These findings suggest that the increase in MBP levels was only found in TgM83 + AAVhSNCA group.

The WB analysis also supported the results of IF findings. Small tissue samples from the striatum were dissected from both the non-injection and the injection sides of the mouse brain. The relative MBP level MBP was calculated by dividing the MBP intensity on the injection side by that of the non-injection side. WB results revealed a 1.62 fold increase in MBP expression on the injection side in Tgm83 + AAVhSNCA group (Fig. 4e, f), compared to WT + AAVhSNCA group (WT + AAVhSNCA: 1.018 ± 0.114 vs. TgM83 + AAVhSNCA: 1.616 ± 0.381, unpaired t-test, *p* = 0.0420) (The corresponding uncropped gels and blots are provided in Supplementary Fig. 1).

Other myelin protein, MOG and PLP, were also measured using IF. Although MOG was increased in WT + AAVhSNCA and TgM83 + AAVhSNCA groups, the difference was not significance (TgM83 + AAVeGFP: 1.198 ± 0.197; WT + AAVhSNCA: 1.082 ± 0.205; TgM83 + AAVhSNCA: 1.321 ± 0.409; *p* = 0.4634). PLP level showed no changes among the groups (TgM83 + AAVeGFP: 0.974 ± 0.071; WT + AAVhSNCA: 0.932 ± 0.143; TgM83 + AAVhSNCA: 0.934 ± 0.244; *p* = 0.9106). The results showed that MBP levels were elevated in AAVhSNCA + TgM83 group, while MOG and PLP levels remained unchanged across all groups (Fig. 4c, d). This suggests that αSyn overexpression in OL, in the presence of pre-existing abnormal (A53T) αSyn, may induce alterations in MBP expression.

### Myelin rearrangement was observed under transmission electron microscopy (TEM)

**Fig. 5:**
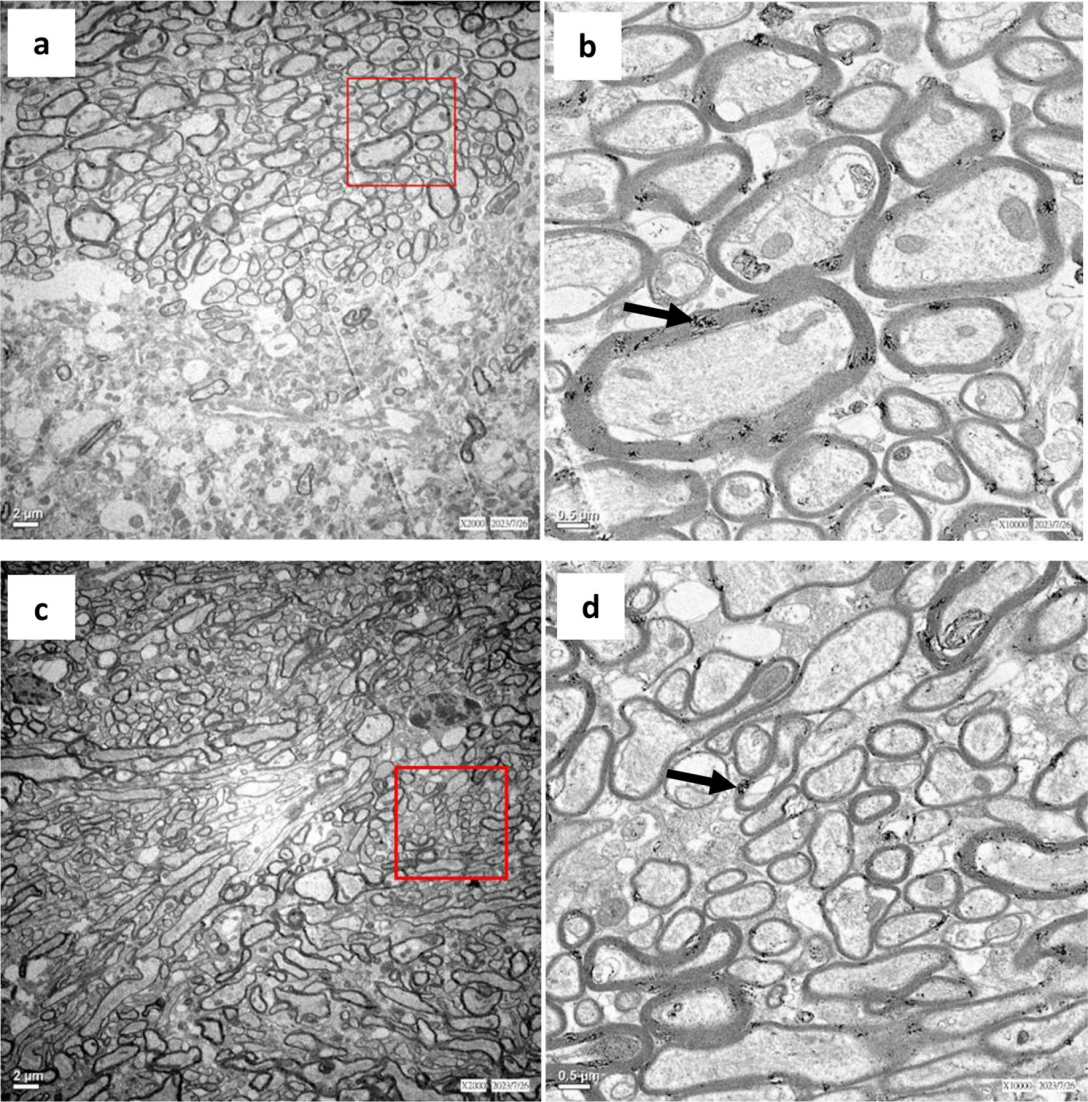
TEM showed robust myelin rearrangement on the injection side of TgM83 + AAVhSNCA mouse. **a**,** b** Non-injection side, scale bar = 2 μm and 0.5 μm. **c**,** d** Injection side, scale bar = 2 μm and 0.5 μm. Multilamellar myelin debris can be seen in the both non-injection and injection sides (**b**,** d**, arrow)

Given the observed elevation of MBP on the injection side, we sought to further examine the underlying alterations through TEM. Extensive white matter rearrangement was observed on the injection side (Fig. 5c, d), but not on the non-injection side, of the TgM83 + AAVhSNCA mouse (Fig. 5a, b). Higher-magnification images revealed disorganized axonal orientation and misalignment. The axons appeared irregular in size, generally smaller, and densely packed. Most of the axons were only surrounded by a thin layer of myelin sheath too. Multilamellar bodies were observed in both the injection (Fig. 5d) and the non-injection side (Fig. 5b), as previously reported [21]. The images suggested that AAVhSNCA could induce prominent white matter rearrangement in TgM83 mice.

### Positive correlation between relative levels of MBP and inflammation responses

**Fig. 6:**
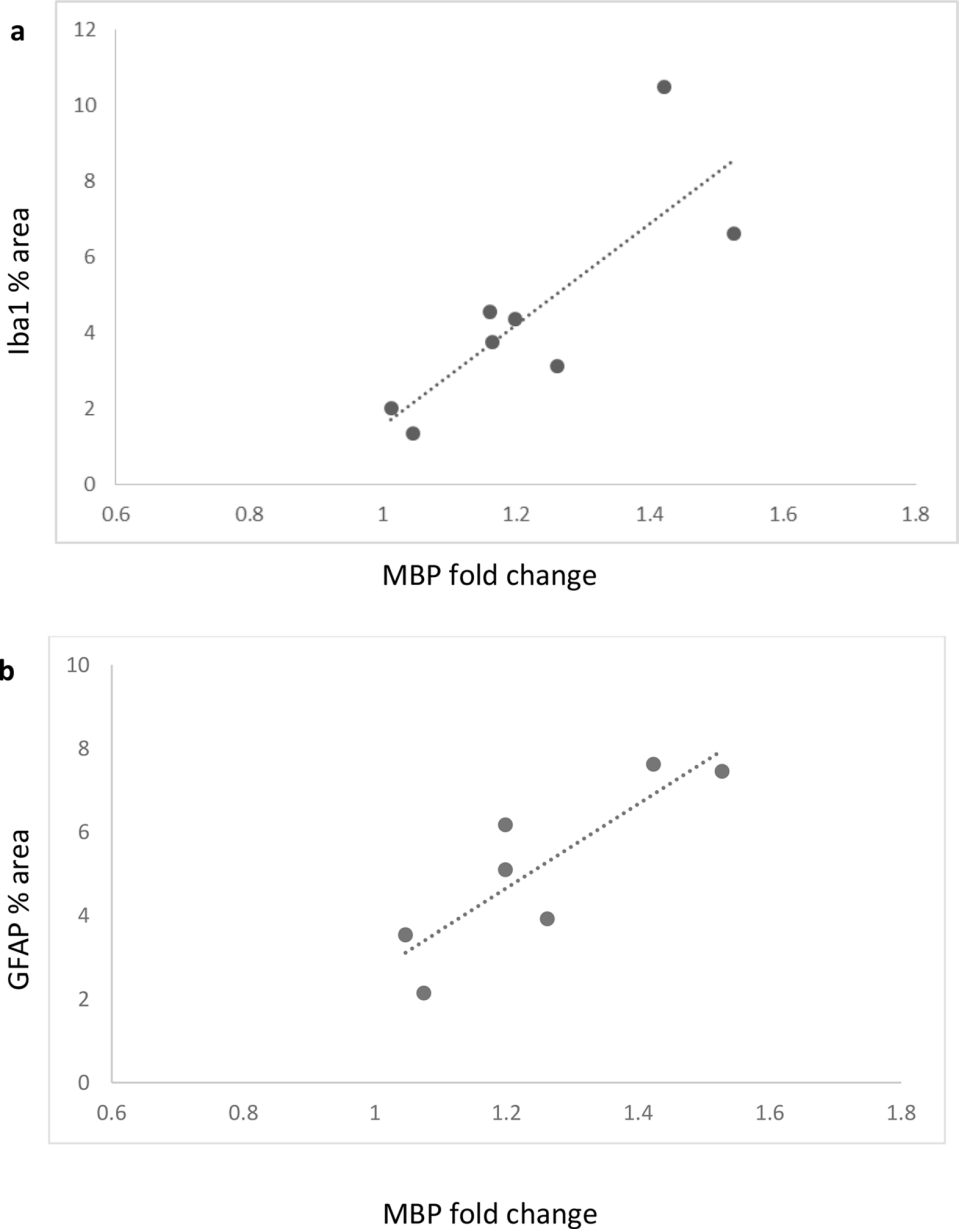
Positive correlation was found between MBP fold change and microglia’s (Iba1) and astrocyte’s activation (GFAP). The data from both WT + AAVhSNCA and TgM83 + AAVhSNCA were included and plotted

Since MBP levels, Iba1 and GFAP were all elevated in TgM83 + AAVhSNCA group, we suspected there could be potential correlation between MBP levels and microglia or astrocyte activation. To examine the relationship between MBP levels and inflammatory responses, the relative MBP levels were plotted against the percent area of Iba1 and GFAP staining (both TgM83 + AAVhSNCA and WT + AAVShSNCA groups were included). Correlation analysis revealed a positive association between MBP expression and the activations of both microglia and astrocytes (Fig. 6a, Iba1% area vs. MBP fold change, correlation = 0.813, *n* = 8, *p* = 0.0141; Fig. 6b, GFAP% area vs MBP fold change, correlation = 0.854, *n* = 7, *p* = 0.0143).

### Post-mortem brain tissue of MSA patient also showed MBP and Iba1 elevation

**Fig. 7:**
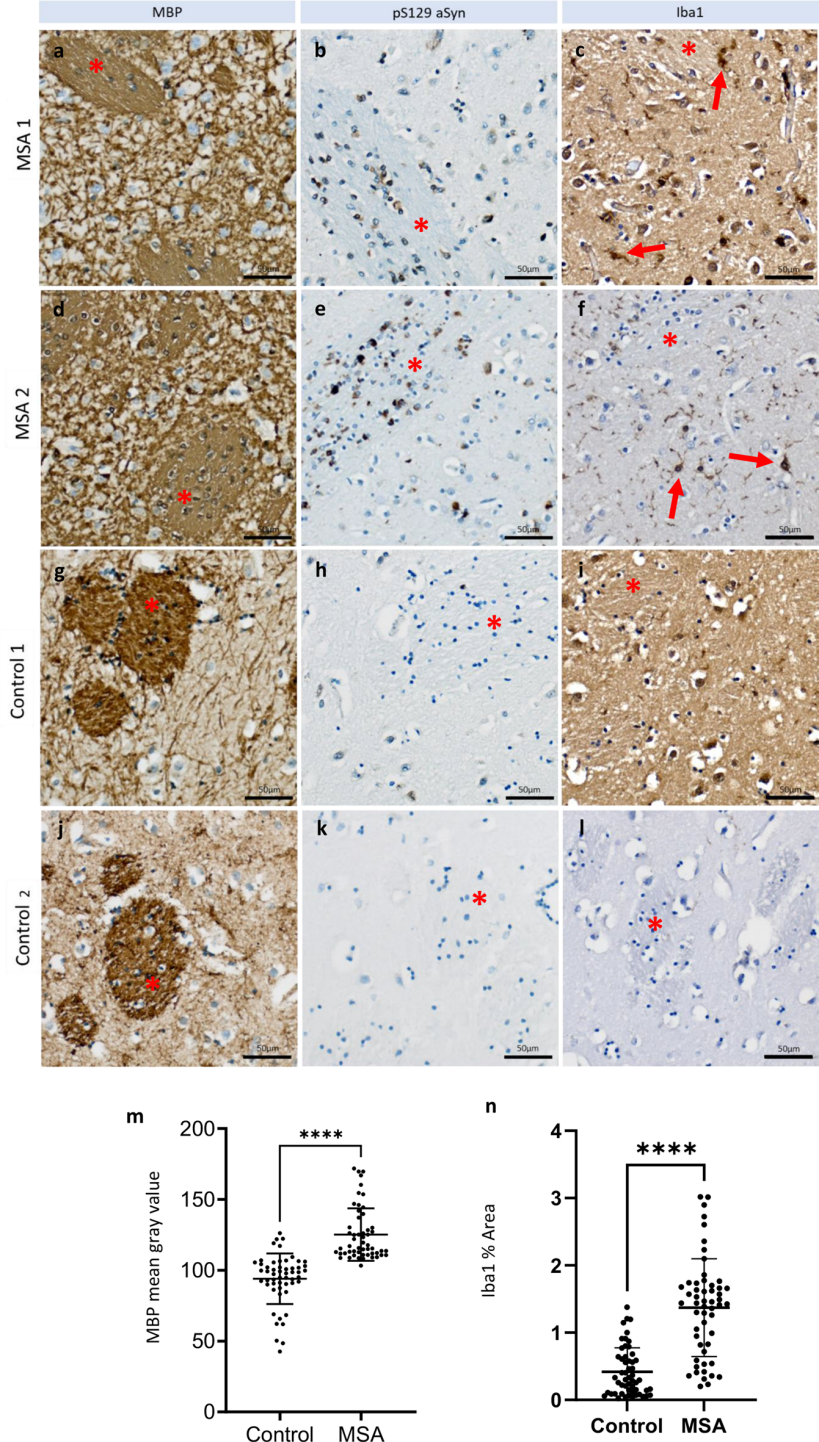
Staining results of MSA patient and control’s post-mortem brain sections using LFB, MBP, pS129 αSyn and Iba1 antibodies. **a**,** d**,** g**,** j** MSA showed higher MBP levels, scale bar = 50 μm. **b**,** e**,** h**,** k** Higher numbers of pS129 αSyn and **c**,** f**,** i**,** l** higher microglia activations (red arrow) compared to control, scale bar = 50 μm. **m** MBP staining revealed higher mean gray value in MSA. **h** Iba1 staining showed that microglia in MSA were larger in size, suggesting they were activated. Star sign (*) indicates striatopallidal fibers or pencil fibers of Wilson), scale bar = 50 μm. **** < 0.0001

Human brain tissue further supports the findings observed in animal models. In post-mortem putamen sections from MSA patients (*n* = 2) and controls (*n* = 2), elevated MBP expression was observed in the MSA sample (Fig. 7a, d, g, j) compared to the control. While the control tissue displayed clear contrast between white and gray matter (Fig. 7g, j), MBP staining in the MSA samples showed comparable intensity in both regions (Fig. 7a, d). Additionally, numerous pS129 α-synuclein-positive cells (Fig. 7b, e) and increased activated microglia (Fig. 7c, f; red arrow) were present in the MSA brain, whereas only sparse pS129 α-synuclein staining (Fig, 7h, k) and minimal microglial activation (Fig. 7i, l) were observed in the control. These results are consistent with observations from the TgM83 + AAV-hSNCA mouse model.

**Fig. 8:**
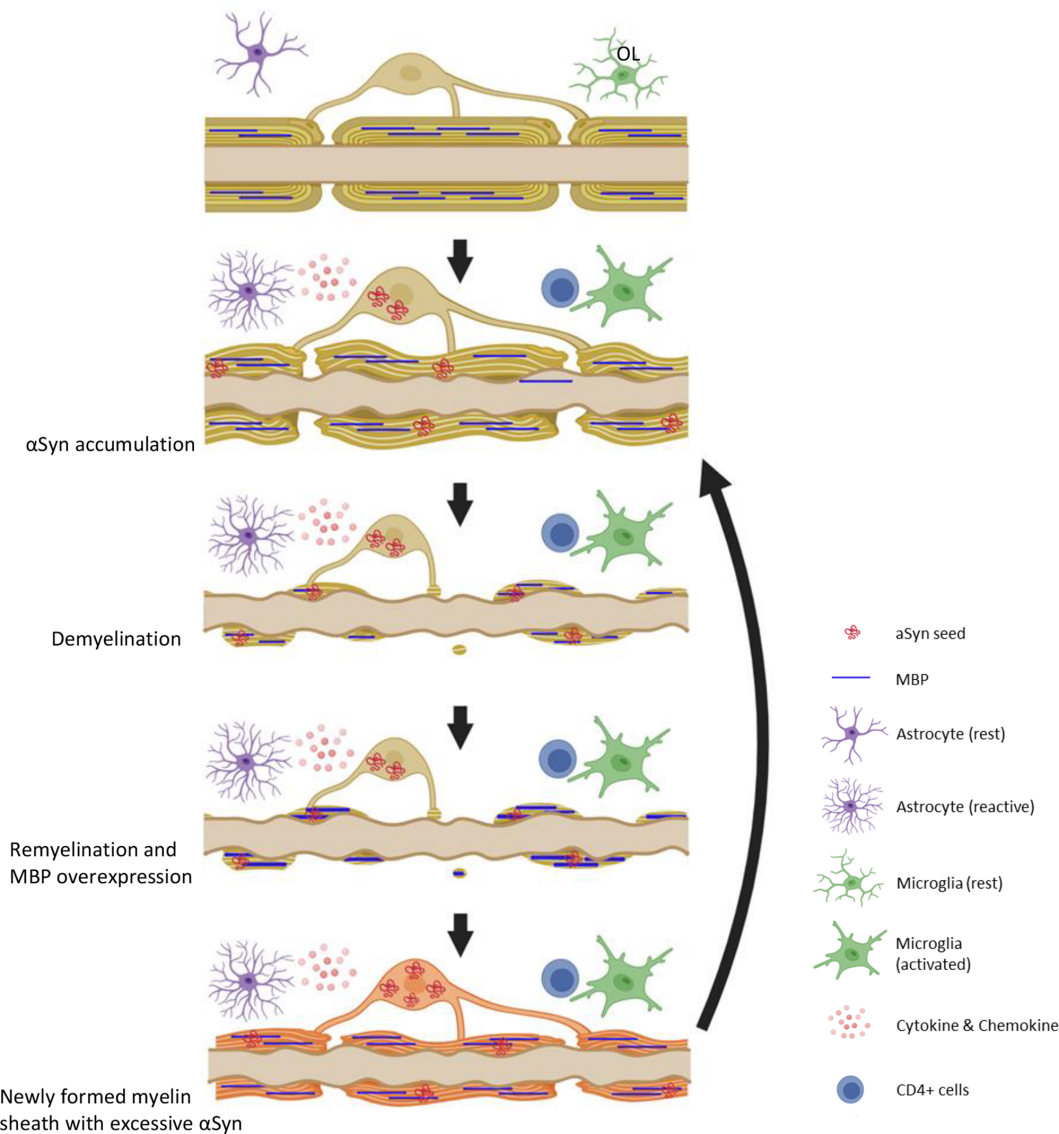
Hypothetical mechanism that illustrates the involvement of microglia and oligodendrocyte (OL) in the process of demyelination and re-myelination in TgM83 + AAVhSNCA mouse model. Top panel: Normal OL, myelinated axon, and microglia. Second panel: alpha synuclein (αSyn) accumulation in OL causes myelin sheath to misfold and inflammation (microglia activation, astrocyte activation, T cell priming, and cytokine secretions). Third panel: Surviving OL starts to degenerate and demyelinate. Microglia is also activated in response to OL damages. Forth panel: Surviving OLs attempt to re-myelinate and expresses more myelin basic protein (MBP). Microglia activation may also participate myelin growth. Fifth panel: newly formed myelin sheath contains pre-existing αSyn, which impair oligodendrocytes’ ability to properly form functional myelin sheaths. Created in http://BioRender.com

Statistical analysis revealed significant difference in MBP intensity between MSA and control (Fig. 8m, control: 94.04 ± 17.85; MSA: 125.2 ± 18.5; *p* < 0.0001). Microglia activation was also observed (Fig, 8n, control: 0.42 ± 0.36; MSA: 1.37 ± 0.73; *p* < 0.0001).

## Discussion

Although there are several animal models that can capture some pathological features of MSA, most of the models either do not selectively induce αSyn overexpression in oligodendrocytes or overexpress αSyn in multiple brain regions concurrently. Thus, making these models less ideal for studying the spreading of αSyn or the disease progression of MSA. The high specificity of this AAVOlig001 induced model offers a unique tool to study how αSyn aggregates and contributes to the developments of MSA pathologies. Our results showed that AAVhSNCA could successfully induce αSyn overexpression in WT and TgM83 mice. We also found that MBP levels increased in the animal model when hSNCA was specifically expressed in the oligodendrocytes in TgM83 mouse line, despite the decreases of overall myelin structure (demyelination). Although AAV-Olig001–induced αSyn overexpression has been shown to replicate MSA-like pathologies in some animal models, such as rodents and nonhuman primates [34, 37], our data suggested that the pre-existence of abnormal (A53T) αSyn can exaggerate the αSyn induced MSA-like pathologies. Most importantly, we found a positive correlation between the levels of MBP and Iba1 activation, suggesting that these processes might be more closely linked than previously thought.

MBP, MOG, and PLP each has different roles in the development of myelin. MBP is the second most abundant protein in CNS myelin (30% of myelin protein) and is responsible for adhesion of the membrane bilayers of myelin membrane structure [9]. The highly positively charged domains of the protein interact with the negatively charged phospholipids and may mediate compaction [51]. An elegant study done by Raasakka and colleagues showed that MBP had important function in the formation major dense line during myelination [44]. Compared to other glycoproteins MOG is only found in relatively small amounts within myelin; however, the outmost external location on myelin sheaths makes it easily accessible to the potential antibodies and T-cell response involvement. PLP is the most abundant myelin protein (50% of myelin protein) [14]. As a structural protein PLP plays a major role in the correct apposition of the extracellular / outer leaflets of the membrane, thereby have important role in stabilizing the intra-period line of myelin membrane structure upon compaction. Whereas MBP is crucial for myelination to occur, PLP appears dispensable [6]. The elevated expression of MBP, but intact MOG and PLP suggested that aberrant myelination process may occur during the formation of major dense line. This may also result in incomplete signal conduction between neurons. This finding is consistent with a recent study which showed that the MBP level was elevated in the CSF of MSA patients compared to PD and controls [47]. Santaella and colleagues collected CSF samples from PD patients (*n* = 55), MSA patients (*n* = 22) and healthy controls (*n* = 118) and measured the concentrations of various proteins [47]. They found that CSF MBP concentrations were increased in MSA compared to PD and controls (*p* < 0.005) and could be used to differentiate MSA and PD at early stage with high accuracy (AUC = 0.781; *p* < 0.001).

On the other hand, some papers suggested that MBP was reduced in MSA patient’s brain. Mészáros and colleagues used a transgenic MSA mouse model that attached hSNCA gene sequence into MBP gene sequence as oligodendrocyte specific promotor, causing αSyn accumulation in OLs [39]. They showed that MBP level was reduced in cerebellum in 8 and 16 weeks old mice, compared to non-transgenic mice. Song and colleagues measured MBP levels in the superior precentral gyrus of 4 MSA patients and controls and found the relative amount of MBP was significantly reduced in MSA [49]. The differences could be due to the different brain regions used by these studies.

We also observed higher microglia and astrocyte activation in injection side of TgM83, compared to WT (Fig. 3). This is consistent with what we observed in human brain sections (Fig. 7). Previous studies also showed that Iba1 and GFAP were increased in MSA compared to control [2]. Locatelli and colleagues showed that strong microglia-macrophage activation was associated with oligodendrocyte death. They treated transgenic mice in which diphtheria toxin receptor is expressed specifically in oligodendrocytes with a low weekly dose of diphtheria toxin for 5 months, and found mild demyelination, sparse ODC death and increased numbers of Iba1^+^ microglia-macrophages in the absence of a clinical phenotype [32]. Cuprizone induced OL cell death had also been shown to trigger leukocyte influx into the CNS [29]. Astroglial cells could respond to CNS injuries and neurodegeneration by undergoing reactive gliosis, a process whereby astroglial cells undergo cellular hypertrophy (increased expression of GFAP) and proliferation (increase number of glial cells) [10, 54].

The majority of the inflammatory markers overlaps with pS129 αSyn signal in both WT and TgM83 mice suggested a strong association between the inflammatory response and αSyn overexpression. Similar finding was found in other studies too. Hoffmann and colleagues used a transgenic mouse model overexpressing human αSyn under the control of a MBP promoter (MBP29-hα-syn mice) and detected an increased inflammation in mouse striatum with a high α-syn load [26]. William and colleagues also injected AAVOlig001 that carries hSNCA into mouse brain and found that oligodendrocyte transfected with this vector resulted in a robust inflammatory response, including microglia activation and infiltration of pro-inflammatory monocytes into the CNS [52].

We also found a pS129 αSyn and TPPP/p25a marked regions both overlap the FLAG marked region (Fig. 1b, c), suggesting the GCIs are likely to form in this region. This is consistent with Ferreira and colleagues’ finding, which stated that oligodendroglial p25α is responsible for generating a highly pro-degenerative αSyn strain in MSA [18].

The exact mechanisms that cause higher levels of inflammation in TgM83 mice, compared to WT, remain unknown. There are several possible explanations. It’s possible that different forms of αSyn are generated in TgM83 + AAVhSNCA and WT + AAVhSNCA. It has been known that pathogenic form of αSyn, such as A53T αSyn, triggers pro-inflammatory microglial response and impairs phagocytosis [24]. The A53T protein also activated microglia more strongly than the wild-type αSyn and other mutants (A30P, E46K) in microglia culture [25]. Murine microglial cell line transfected with A53T αSyn also demonstrated increased secretion of the proinflammatory cytokines, tumor necrosis factor-a (TNF-a) and interleukin-6 (IL-6), as well as increased nitric oxide production [45]. One possible reason that explain why WT + AAVhSNCA does not generate extensive inflammation is because the soluble form of αSyn. A study done by Laferrière and colleagues found that most the phosphorylated αSyn found in the brains of the PLP-hSNCA mice (one of the transgenic mouse models of MSA) are mostly soluble (> 80%) and does not recapitulate the fibrillar aggregated form of αSyn in MSA [31]. This may explain why the increased level of pS129 αSyn in WT does not lead to enhanced Iba1 activation.

The cytokine profile provides a detailed view of what happed in the injection side in TgM83 compared to the non-injection side. We found that IL-1 α, CCL2, CCL4, IL-10, IL-12(p40), CCL3 and CCL5 were significantly increased in injection side of TgM83 mice. These cytokines possess both pro-inflammatory (IL-1 α, IL-12(p40), CCL2) and anti-inflammatory (IL-10) effects, suggesting a complex pro- and anti-inflammatory interaction may exist. It has been found that microglia are able to switch between pro and anti-inflammatory states and interact with neurons, astrocytes and oligodendrocytes in order to maintain tissue homeostasis in response to the environment [27]. Some of these molecules are consistent with previous finding which showed that IL-10, IL-12(p40), and CCL4 (MIP-1β) were significantly higher in MSA vs. non-MSA cases [11]. CCLs can stimulate the movement and the migration of leukocytes [36], and IL-12(p40) plays an important link between innate and adaptive immunity [1]. These molecules represent important cues for recruiting adaptive immune system near the injected regions. Our study showed the number of CD4^+^ cells was increased in the injection side (Fig. 3g, h). This suggested that innate immune cells, as well as adaptive immune system, may be modulated [33]. This is also consistent with previous finding that T cell priming and infiltration into the CNS are key mechanisms of disease pathogenesis in MSA [52] and PD [23].

In this study, we also found positive correlations between the relative levels of MBP and microglia’s and astrocyte’s activation (Fig. 6), this not only suggested that MBP level and inflammation response are both closely associated with αSyn overexpression, but also indicated that functional associations may exist between myelination and inflammation during the pathogenesis of MSA. In fact, previous studies have also shown that oligodendrocyte and microglia do not function independently. McNamara and colleagues used transgenic mice without microglia and demonstrated that microglia were required for regulating myelin growth, preventing myelin degeneration, and preserving myelin integrity [38]. Similarly, Miron and colleagues showed that microglia activated by IL-2 and IL-10 could enhance the survival and differentiation of OPCs [40]. In another study, Boccazzi and colleagues utilized purified primary OPC cultures and demonstrated that in vitro TLR3 activated PDGFRα^+^ OPCs significantly increased the release of CCL2, CCL3, CCL5 and CCL11 in their culture medium [7, 8]. These findings suggest that mutual communication may exist between oligodendrocyte and microglia. Interestingly, CCL2, CCL3 and CCL5 were also elevated in our data (Fig. 3).

Although the correlation between synucleinopathy, demyelination, and inflammation in the pathogenesis of MSA is well recognized [17, 55], the changes in myelin proteins have received less attention. It remains unclear whether αSyn overexpression primarily hinders the initiation of remyelination in the early stages, or disrupts OL maturation and myelin sheath formation during later phases of the remyelination process. Our findings suggest that OLs with elevated αSyn levels retain the capacity to initiate remyelination by upregulating MBP expression. This finding aligns with MBP staining observed in post-mortem brain tissue from MSA patients. These results indicate that MBP upregulation may have compensatory role in rescuing or restoring myelin sheath loss in response to αSyn overexpression and inflammation. Furthermore, sustained MBP overexpression in human brain samples during advanced stages of MSA suggests that the formation of stable and mature myelin sheaths may be impaired, or that the rate of remyelination is insufficient to keep up with ongoing myelin loss, prompting OLs to continuously attempt to remyelinate.

Based on these observations, we propose a hypothetical model of MSA pathogenesis (Fig. 8). In the early phase, excessive αSyn accumulation in OLs leads to the formation of insoluble aggregates, contributing to OL dysfunction and demyelination. This loss of myelin integrity subsequently triggers inflammatory responses, including microglial and astrocytic activation [56]. Recent studies suggest that OLs that survive demyelination are capable of extending new processes and re-engaging in remyelination [5, 16, 19, 41]. During remyelination process, MBP is upregulated to support proper myelin ensheathment. However, residual or reabsorbed αSyn during the remyelination process may further compromise OL viability, perpetuating inflammation and leading to incomplete or unstable myelin formation. Ultimately, this cycle of abnormal αSyn accumulation, inflammation, and attempted remyelination may lead to the progressive pathology observed in MSA.

However, some limitations exist. First, pronounced neuronal loss was not observed in our study [37]. This discrepancy may be attributed to the relatively low viral titer used in our experiments. Despite the lower titer, we maintained consistent injection volumes across treatment and control groups to minimize the risk of mechanical damage from excessive fluid infusion. It’s expected that higher titer virus or multiple injections at striatum and substantia nigra would result in more substantial neuronal loss and greater upregulation of MBP expression. Nonetheless, as the primary objective of this study was to assess changes in myelin-associated proteins and inflammatory responses in the context of αSyn overexpression, the limited neuronal loss does not compromise the interpretation of our findings. Second, although previous studies have reported elevated levels of pro-inflammatory cytokines such as TNF-α, IL-1β, and IL-6 in the CSF of MSA patients compared to those with Parkinson’s disease [50], we did not detect similar cytokine increases in our mouse model. This discrepancy may reflect interspecies differences (human vs. mouse) or differences in disease stage, as our model did not exhibit the extensive neuronal loss characteristic of advanced MSA pathology [42].

In summary, although several studies have demonstrated interactions between oligodendrocytes and microglia, and the relationship between α-synuclein and inflammation is well established, to our knowledge, this study is the first to reveal a positive correlation among synucleinopathy, myelination, and inflammation. Our findings suggest that MBP may play an important role in the pathogenesis of MSA and highlight the potential of targeting remyelination pathways, particularly MBP regulation, as a novel therapeutic approach to modifying disease progression.

## Electronic supplementary material

Below is the link to the electronic supplementary material.

Supplementary Material 1

Supplementary Material 2

Supplementary Material 3

Supplementary Material 4

## Data Availability

The datasets used and/or analysed during the current study available from the corresponding author on reasonable request.

## Abbreviations

AAVeGFP: AAVOlig001/PscAAV-CBh-FLAG-EGFP
AAVhSNCA: AAVOlig001/PscAAV-CBh-FLAG-hSNCA
αSyn: alpha-synuclein
CCL: CC chemokine ligand
CRP: C-reactive protein
CSF: Cerebrospinal fluid
CXCL1: Chemokine 1
FFPE: Formalin-fixed paraffin-embedded
FGF-2: Fibroblast growth factor 2
GCI: Glial cytoplasmic inclusions
G-CSF: Granulocyte colony-stimulating factor
GFAP: Glial fibrillary acidic protein
GM-CSF: Granulocyte-macrophage colony-stimulating factor
Iba1: Ionized calcium-binding adapter molecule 1
IF: Immunofluorescence
IFN-γ: Interferon gamma
IHC: Immunohistochemistry
IL: Interleukin
INF-a2: Interferon alpha-2
LFB: Luxol fast blue
MBP: Myelin basic protein
MIP-1β: Macrophage inflammatory protein
MOG: Myelin oligodendrocyte glycoprotein
MSA: Multiple system atrophy
OL: Oligodendrocytes
OPC: Oligodendrocyte progenitor cells
PD: Parkinson’s disease
PLP: Proteolipid protein
pS129 αSyn: Phosphorylated alpha-synuclein at S129
PSP: Progressive supranuclear palsy
TEM: Transmission electron microscopy
TNF-α: Tumor necrosis factor alpha
TPPP/p25a: Tubulin polymerization-promoting protein
VEGF: Vascular Endothelial Growth Factor
