# Supplementary material for: Oligodendrocyte-specific overexpression of human alpha-synuclein results in elevated MBP levels and inflammatory responses in TgM83 mice, mimicking the pathological features of multiple system atrophy
Source: Acta Neuropathol Commun. 2025 May 7;13:94. doi: 10.1186/s40478-025-02014-y (PMC12060544; doi:10.1186/s40478-025-02014-y)
Supplement: Supplementary file 1 — Supplementary Material 1 [file 40478_2025_2014_MOESM1_ESM.docx]

Supplementary Fig. 1


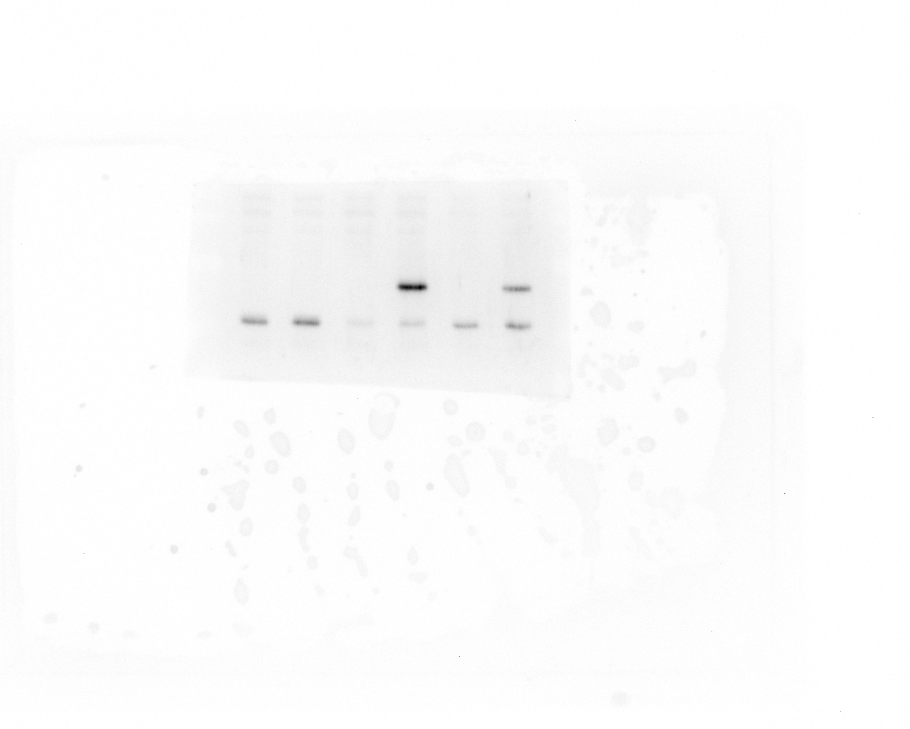

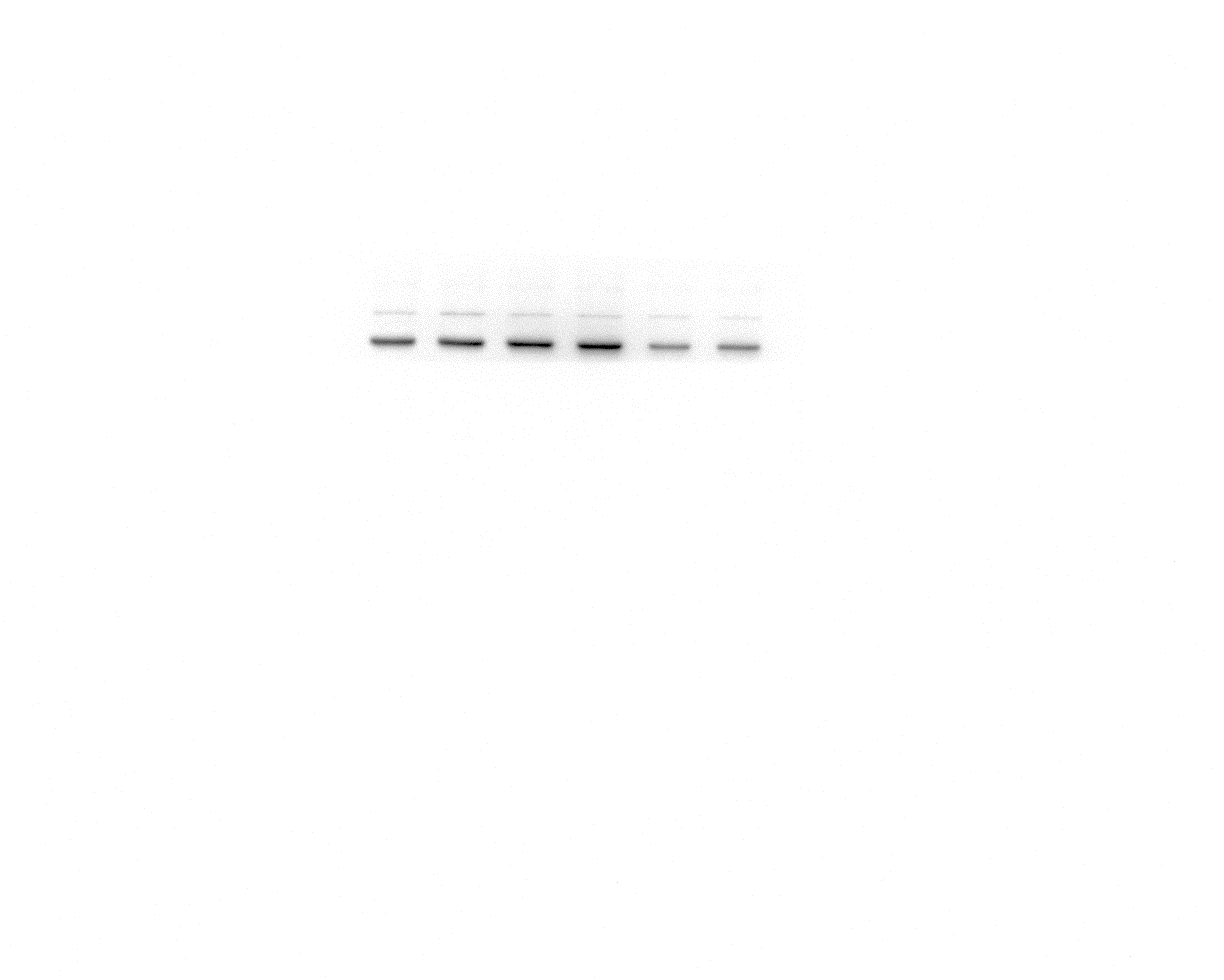


**a**

**b**


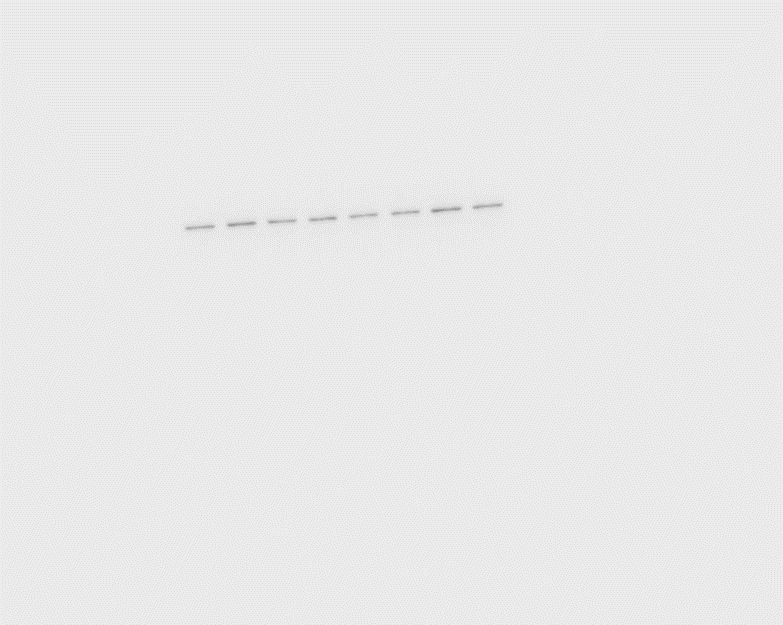


**c**


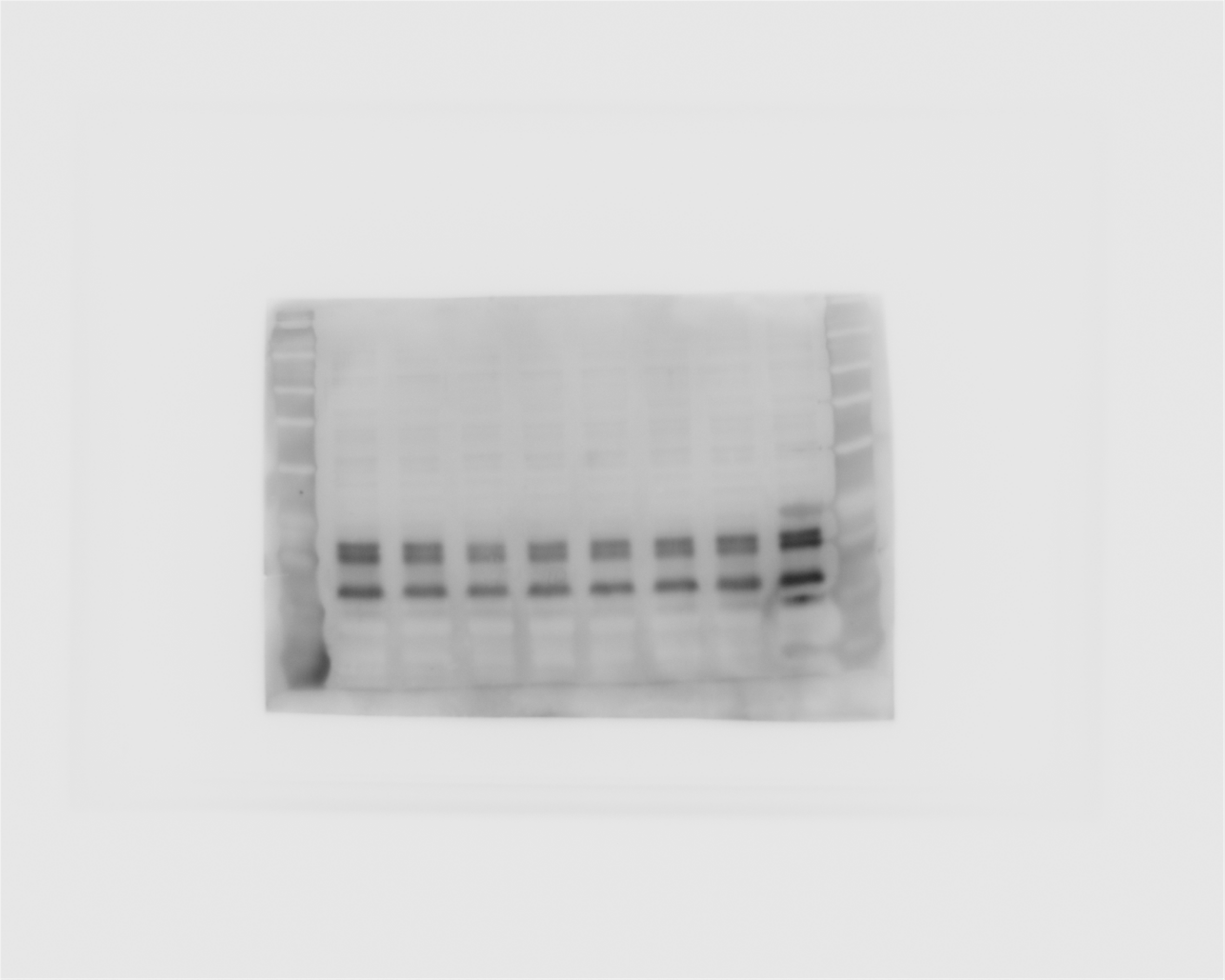


**d**

**Supplementary Fig. 1** The uncropped Gels and Blots images. The dotted line marked the cropped regions used in the main test. **a** Corresponding to figure 1 d, anti-tubulin, Genetex GTX101279 (1:10000), was used. **b** Corresponding to figure 1 d, recombinant anti-alpha-synuclein (phospho S129) antibody [EP1536Y], Abcam ab51253 (1:5000), was used. **c** Corresponding to figure 5 e, anti-HSP60, abcam 45134(1:10000), was used. **d** Corresponding to figure 5 e, anti-MBP, clone SMI 99, purified, BioLegend (1:5000), was used.
