# Supplementary material for: Oligodendrocyte-specific overexpression of human alpha-synuclein results in elevated MBP levels and inflammatory responses in TgM83 mice, mimicking the pathological features of multiple system atrophy
Source: Acta Neuropathol Commun. 2025 May 7;13:94. doi: 10.1186/s40478-025-02014-y (PMC12060544; doi:10.1186/s40478-025-02014-y)
Supplement: Supplementary file 2 — Supplementary Material 2 [file 40478_2025_2014_MOESM2_ESM.docx]

Supplementary Fig. 2

**The number of NeuN-positive cells**

**b**

**a**

**Supplementary Fig. 2** The number of NeuN-positive cells on the injection side in (**a)** WT and (**b)** TgM83 mice injected with saline, AAV-eGFP, or AAV-hSNCA. No significant differences in neuronal density (cells/mm²) were observed among the groups.
