# Supplementary material for: Oligodendrocyte-specific overexpression of human alpha-synuclein results in elevated MBP levels and inflammatory responses in TgM83 mice, mimicking the pathological features of multiple system atrophy
Source: Acta Neuropathol Commun. 2025 May 7;13:94. doi: 10.1186/s40478-025-02014-y (PMC12060544; doi:10.1186/s40478-025-02014-y)
Supplement: Supplementary file 3 — Supplementary Material 3 [file 40478_2025_2014_MOESM3_ESM.docx]

Supplementary Fig. 3

**Behavioural test: method**

Rotarod, pole test and open field test were used to access the mice’s motor functions two months after injections.

Rotarod. TgM83+AAVhSNCA, n = 8; TgM83 hemi, n = 11; TgM83+AAVeGFP, n = 3. Animals were put on the rod (UGO BASILE Rota-Rod 47600) at 4rpm for at least one minute before testing. Mice were trained for 3 consecutive days before the testing day. Mice stayed on the rod for 300 secs during training. The speed was increased each day; day 1 at 6rpm, day 2 at 12 rpm, and day 3 at 18 rpm. During the test, the rod was accelerated from 4 to 40 rpm over 300 sec. The latency to fall was recorded for each trial, and the average of three trials was reported.

Pole test: Mice (groups, n = 3) are placed in the top of a 60 cm vertical pole with a diameter of 1 cm. The pole was wrapped with adhesive tape to increase the friction. The pole was placed in the home cage so that mice might prefer to descend to the floor of cage. Recording start when the animal start to descend. Mice were placed with the head down at the top of the pole and the total time for climbing down the pole until the mouse reached the floor with the four paws was taken in 3 trials. The average of all 3 trials was used for the statistical analysis.

Open field: Locomotor activity will be tested using the open-field test. Mice will be placed individually in the middle of a test chamber (40cm x 40 cm; Med Associates, Inc., Fairfax, VT) and allowed to explore the arena for 10 min over 3 trials. The average of all three trials was used for the statistical analysis. Movement was recorded using a camera from above. Data was collected by the Exovision software. The mouse’s head and tail will be identified in the software and tracked throughout the trial. The following parameters will be measured: total distance travelled (mm), speed (m/s) and time in the center area (20 x 20 cm) were recorded for analysis.

The Student’s t-test (unpaired) was used to compare the behavioural differences, inflammation proteins and WB of MBP.

**Behavioural test: result**

No motor dysfunction was detected in TgM83+AAVhSNCA.

Rotarod, pole-test and open field test were also used to evaluate the mice’s motor functions. However, none of the behaviour tests showed significant difference. Rotarod did not reveal significant difference in the times that the mice stayed on the rod among TgM83 groups (supplementary figure S2 **a**; TgM83 hemi: 190.8 ± 61.25, TgM83+eGFP: 246.9 ± 50.35; TgM83+hSNCA: 213.5 ± 75.77, one-way ANOVA, p = 0.4162). Pole-test did not show difference between the time taken by TgM83+AAVeGFP and TgM83+AAVhSNCA to travel from the top to the bottom of the pole (supplementary figure S2 **b**; TgM83+AAVeGFP: 9.23 ± 1.631, TgM83+AAVhSNCA: 7.573 ± 3.886; unpaired t test, p = 0.5333). Open-field test did not show any significant different in travel distance and time in zones either (WT and TgM83+AAVhSNCA, n = 2 in each group, data not shown). These results indicated that partial demyelination in the striatum did not induce phenotypic motor deficits in the mice.


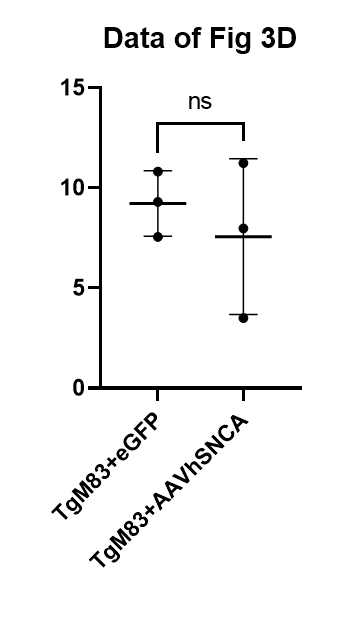

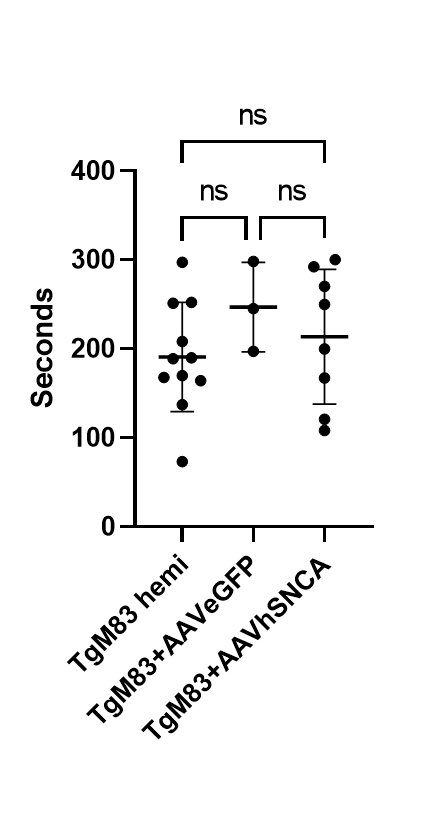


Pole test

Seconds

Rotarod

Seconds

**b**

**a**

**Supplementary Fig. 3** The behaviour tests did not reveal significant differences in TgM83+AAVhSNCA mice. **a,** rotarod test; **b,** pole-test.
