# Supplementary material for: Oligodendrocyte-specific overexpression of human alpha-synuclein results in elevated MBP levels and inflammatory responses in TgM83 mice, mimicking the pathological features of multiple system atrophy
Source: Acta Neuropathol Commun. 2025 May 7;13:94. doi: 10.1186/s40478-025-02014-y (PMC12060544; doi:10.1186/s40478-025-02014-y)
Supplement: Supplementary file 4 — Supplementary Material 4 [file 40478_2025_2014_MOESM4_ESM.docx]

Supplementary Table 1

| Cytokine and chemokine list | Non-injection side  Average | SD | Injection side  Average | SD | P value  Paired, one-tail |
| --- | --- | --- | --- | --- | --- |
| IL-1 α | 5.69 | 0.55 | 8.03 | 0.5 | 0.01 |
| IL-1 β | 1.05 | 0.23 | 1.18 | 0.06 | 0.25 |
| IL-2 | 65.89 | 21.2 | 73.52 | 15.37 | 0.35 |
| IL-3 | 1.09 | 0.14 | 1.07 | 0.17 | 0.46 |
| IL-4 | 0.34 | 0.11 | 0.4 | 0.15 | 0.33 |
| IL-5 | 0.95 | 0.3 | 0.98 | 0.41 | 0.47 |
| IL-6 | 2.21 | 0.62 | 2.3 | 0.49 | 0.44 |
| IL-9 | 5.44 | 1.6 | 4.81 | 0.81 | 0.32 |
| IL-10 | 13.91 | 2.11 | 23.71 | 2.23 | 0.01 |
| IL-12(p40) | 11.3 | 2.65 | 277.15 | 143.55 | 0.03 |
| IL-12(p70) | 15.91 | 2.23 | 15.53 | 4.70 | 0.46 |
| IL-13 | 21.66 | 7.10 | 22.99 | 7.38 | 0.43 |
| IL-17A | 3.53 | 1.43 | 3.42 | 1.38 | 0.47 |
| Eotaxin/CCL11 | 3.84 | 1 | 4.50 | 0.42 | 0.22 |
| G-CSF | 2.72 | 0.23 | 2.82 | 0.18 | 0.33 |
| GM-CSF | 13.46 | 1.2 | 12.21 | 1.46 | 0.20 |
| IFN-γ | 6.91 | 1.25 | 6.31 | 1.03 | 0.31 |
| KC/ Gro-α/ CXCL1 | 2.58 | 1.25 | 3.01 | 0.42 | 0.34 |
| MCP-1/ MCAF / CCL2 | 66.30 | 13.42 | 121.69 | 8.01 | <0.01 |
| MIP-1α/ CCL3 | 1.38 | 0.33 | 8.72 | 3.80 | 0.03 |
| MIP-1β/ CCL4 | 18.54 | 3.51 | 40.27 | 5.99 | 0.01 |
| RANTES/CCL5 | 29.72 | 3.68 | 76.44 | 26.77 | 0.04 |
| TNF-α | 15. | 1.71 | 15.33 | 1.07 | 0.42 |

**Supplementary Table 1** Bio-plex immunoassays results. Mean ± SD, unit = ng/ml.
